# High-grade soft tissue sarcoma arising in a desmoid tumor: case report and review of the literature

**DOI:** 10.1186/s13569-015-0040-0

**Published:** 2015-11-30

**Authors:** François Bertucci, Marjorie Faure, Maria-Rosa Ghigna, Bruno Chetaille, Jérôme Guiramand, Laurence Moureau-Zabotto, Anthony Sarran, Delphine Perrot

**Affiliations:** Department of Medical Oncology, Centre de Recherche en Cancérologie de Marseille (CRCM), Institut Paoli-Calmettes, INSERM UMR1068, CNRS UMR725, 232 Bd de Sainte-Marguerite, 13009 Marseille, France; Faculty of Medicine, Aix-Marseille University, Marseille, France; Department of Pathology, Marie Lannelongue Surgical Centre, Le Plessis-Robinson, France; Department of Pathology, Institut Paoli-Calmettes, Marseille, France; Department of Surgical Oncology, Institut Paoli-Calmettes, Marseille, France; Department of Radiotherapy, Institut Paoli-Calmettes, Marseille, France; Department of Radiology, Institut Paoli-Calmettes, Marseille, France

**Keywords:** Breast cancer, Desmoid tumor, Soft tissue sarcoma

## Abstract

Desmoid tumors are rare benign monoclonal fibroblastic tumors. Their aggressiveness is local with no potential for metastasis or dedifferentiation. Here we report on a 61-year-old patient who presented a locally advanced breast desmoid tumor diagnosed 20 years after post-operative radiotherapy for breast carcinoma. After 2 years of medical treatment, a high-grade undifferentiated pleomorphic soft tissue sarcoma arose within the desmoid tumor. Despite extensive surgery removing both tumors, the patient showed locoregional relapse by the sarcoma, followed by multimetastatic progression, then death 25 months after the surgery. The arising of a soft tissue sarcoma in a desmoid tumor is an exceptional event since our case is the fourth one reported so far in literature. It reinforces the need for timely and accurate diagnosis when a new mass develops in the region of a preexisting desmoid tumor, and more generally when a desmoid tumor modifies its clinical or radiological aspect.

## Background

Desmoid tumors (DT) are rare benign monoclonal fibroblastic tumors representing less than 5 % of all soft tissue tumors [1]. Also called aggressive deep-seated fibromatosis, they typically arise from muscular or aponeurotic structures. Their location may be extra-abdominal [2] or in the abdominal wall, usually sporadic, or intra-abdominal [3], often associated with familial adenomatosis polyposis (FAP) [1]. The mammary location is very rare (<10 % of all cases), the literature being limited to case reports and three retrospective series totalizing 81 patients [4–6]. Such location does not seem to differ in term of clinical outcome than other locations.

Desmoid tumors grow slowly. Their aggressiveness is local with no potential for metastasis or dedifferentiation. In the last 2012 World Health Organization (WHO) classification of sarcomas, they are classified as tumors with intermediate malignancy potential. Locally, they can damage vital structures and threaten life [7], notably in intra-abdominal locations, and have a high rate of recurrence even after complete resection. Their treatment remains challenging, based on diverse strategies [7, 8]: “wait-and-see policy”, non-steroidal anti-inflammatory drugs (NSAIDs), hormone therapy, chemotherapy and recently investigational targeted therapies (imatinib, sorafenib, pazopanib), and surgery and/or radiation therapy. Their natural history is not well-defined and poorly understood, including long periods of stabilization, or even spontaneous regression.

The arising of a soft tissue sarcoma in a DT is an exceptional event, with only three cases reported to date [9–11]. Here we report on a 61-year patient who presented a locally advanced breast DT refractory to multiple systemic therapies and in which arose a rapidly lethal high-grade undifferentiated soft tissue sarcoma.

## Case presentation

### Description of the case

In April 2010, a 61-year-old woman, Caucasian type, presented inflammation of the right breast with induration and palpable mass in its medial part. The biopsy was negative. In October 2010, the breast MRI showed an 8-cm tumor infiltrating the whole mammary gland and the pectoralis muscle, with enhancement after injection and skin thickening. The biopsy showed an “extensive and collagenic extensive fibrosis”. The patient was thus referred to our institution in December 2010.

**Fig. 1:**
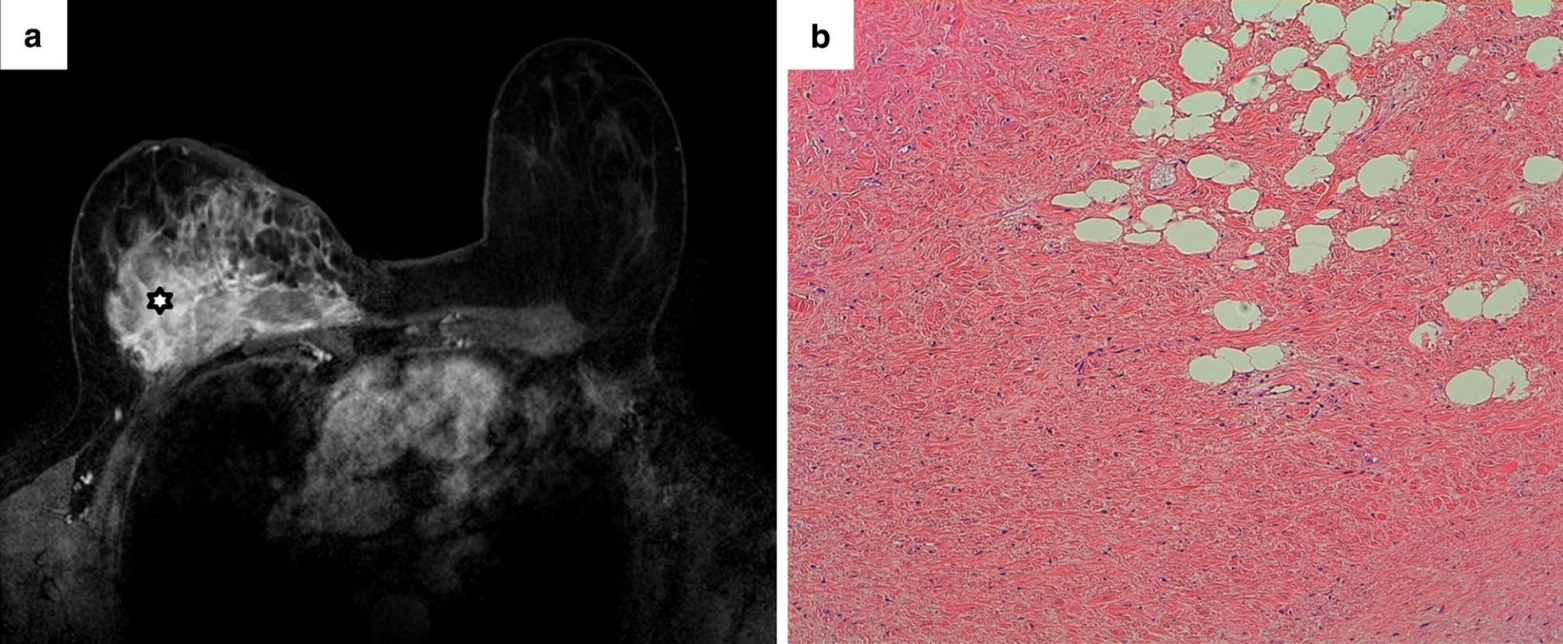
Mammary desmoid tumor: radiological and pathological aspects. **a** Breast MRI of January 2011: T1-weighted Fat Sat images of the *right* breast after gadolinium injection. The white star shows the desmoid tumor. **b** Microscopic aspect (hematoxylin and eosin staining, HES) of the diagnostic biopsy showing a poorly cellular proliferation of bland elongated cells in a collagen-rich stroma. Proliferation infiltrates adipose tissue (*right upper corner*)

The clinical examination showed a firm, 8-cm mass, in the medial part of the right breast, poorly circumscribed. There was no palpable axillary and supra-clavicular lymph node. The left breast was normal. The WHO performance status was equal to 0. No history of personal or familial adenomatous polyposis or DT was present, but she had been treated 20 years earlier for an in situ lobular adenocarcinoma of right breast with conservative surgery and adjuvant radiotherapy (60 Gy/30 fractions). Breast MRI revealed spread infiltration of breast and pectoralis muscle (Fig. 1a). An ultrasound-guided biopsy was done. Pathological analysis (Fig. 1b) revealed a poorly cellular proliferation of myofibroblastic spindle cells, without histological evidence of malignancy (no atypia, no mitosis, no necrosis). Immunohistochemistry (IHC) showed strong nuclear expression of β-catenin by tumor cells, but no expression of desmin, H-caldesmon, S100 protein, MDM2, CDK4, AE1/AE3 cytokeratins and hormonal receptors. Histology was reviewed within the French Sarcoma Network (*Réseau de Référence en Pathologie des Sarcomes, RRePS*) and the diagnosis of mammary DT was retained, confirmed by the evidence of β-catenin exon 3 T41A mutation.

Given the tumor volume, no surgery was performed. A first-line systemic treatment combining celecoxib and high-dose tamoxifen was instituted. After 3-month treatment, the tumor progressed and a second-line treatment based on polychemotherapy (doxorubicin-dacarbazine) was delivered for 6 cycles, leading to disease stabilization. Imatinib-based third-line treatment was started on December 2011 because of disease progression, but prematurely stopped because of digestive toxicity. On February 2012, the disease locally progressed: a fourth-line treatment based on the intra-venous methotrexate-vinblastine combination was started without any clinical benefit after 3-months treatment. Aromatase inhibitor was delivered from May 2012. Due to disease progression on September 2012, sorafenib treatment was started, quickly stopped because of major skin toxicity, and thus replaced by sunitinib leading to disease stabilization during 2 months.

**Fig. 2:**
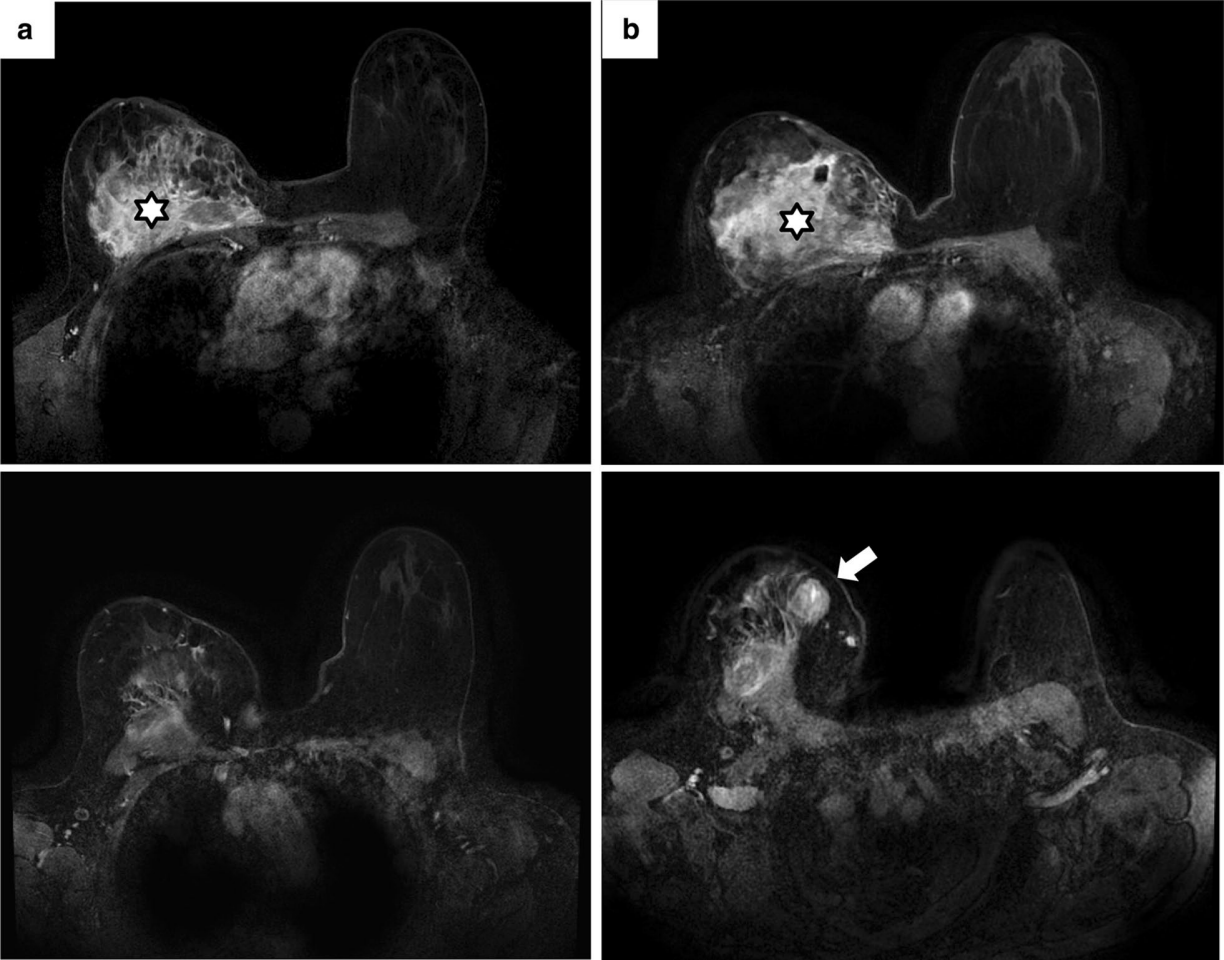
Appearance of a tumor within the desmoid tumor: radiological aspect. Breast T1-weighted Fat Sat MR imaging of middle (*top*) and upper (*bottom*) sections of the *right* breast after gadolinium injection in January 2001 (**a**) and in January 2013 (**b**). The desmoïd tumor (*white star*) increased in size between the two dates. Surprisingly, a 2-cm tissue mass (*white arrow*), corresponding to an undifferentiated sarcoma, appeared in the *upper-inner* quadrant within the desmoid tumor in January 2013

**Fig. 3:**
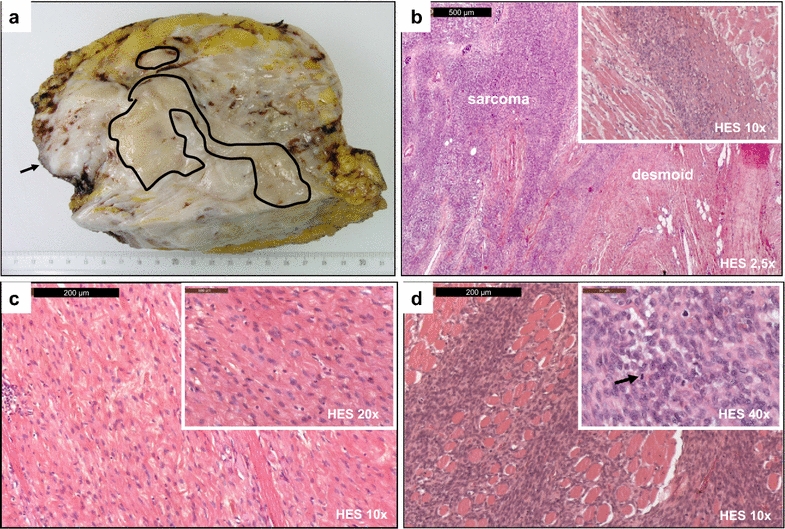
Pathological aspect of the surgical specimen containing the desmoid tumor and the sarcoma. **a** Macroscopic aspect: an ill-defined fibrous-like area occupies most of the specimen, with contact to resection margin (*arrow*). Within this lesion two less fibrous areas are identified (*black lines*). These ones correspond to high-grade sarcoma zones on microscopic examination. **b** Microscopic aspect (HES) showing abrupt transition between high grade sarcoma (*left*) and DT (*right*). **c** Microscopic aspect (HES) showing the DT: spindle cell proliferation of fibroblastic-like cells, interspersed within a collagenous matrix. Spindle cells are void of cytological atypia, and no mitosis or necrosis are seen. **d** Microscopic aspect (HES) showing the high grade sarcoma area. The neoplastic proliferation is more cellular and dense. Spindle cells are disposed in sheets infiltrating the skeletal muscle bundles. They appear small with indistinct cytoplasmic limits; nuclei are oval or round and display dense chromatin. Mitoses are easily found (*black arrow*)

On January 2013, the tumor modified its clinical aspect with further volume increase and appearance of a multilobulated aspect with a fine and tense skin over the tumor lobules, suggestive of a likely underlying sarcoma. The performance status was equal to 1. Breast MRI showed the appearance of a 2-cm tissue mass in the upper-inner quadrant within the DT (Fig. 2). Surgery was decided. On February 2013, the patient underwent a wide chest wall surgical resection including the whole right breast, the third to sixth right ribs, the right half of sternum associated with reconstruction. The macroscopic pathological report described a white mass with a hard consistency, an aspect sometimes fasciculated, realizing subcutaneous nodules (Fig. 3a). An ill-defined fibrous-like area occupied most of the specimen, with contact to resection margin. Within this lesion, two less fibrous areas were identified. Microscopic analysis revealed two lesions (Fig. 3b–d): the DT, with spindle cell proliferation of fibroblast-like cells, without atypia, without mitosis, nor necrosis, interspersed within a dense collagenous matrix, contrasting with a high-grade sarcoma in the two less fibrous areas showing a more cellular and dense proliferation of spindle cells disposed in sheets infiltrating the skeletal muscle bundles, with indistinct cytoplasmic limits, atypia and numerous mitoses. Resection margins were tumor-free for sarcoma, but involved for DT. No adjuvant treatment was delivered.

**Fig. 4:**
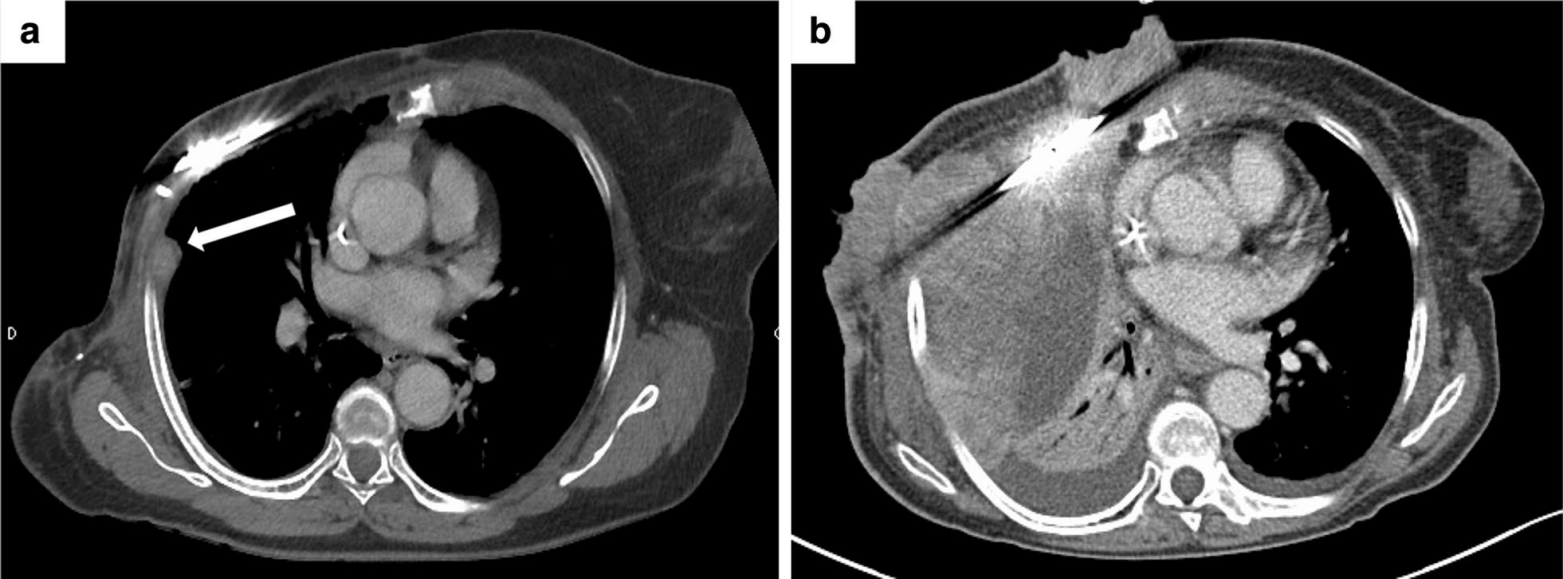
Relapse of the high-grade sarcoma: radiological aspect. **a** CT-scan in June 2013 showed the appearance of a 2-cm right pleural nodule in the operative field (*white arrow*). The percutaneous biopsy achieved 2 months later will identify high-grade undifferentiated spindle-cell sarcoma. **b** Major disease progression in September 2014, involving the pleura, the *right* anterior chest wall and the liver

During follow-up, on June 2013, the CT scan showed the appearance of a suspect 2-cm pleural nodule in the operative field (Fig. 4a). The nodule increased to 5 cm on August and was biopsied, showing relapse by a high-grade undifferentiated spindle-cell sarcoma with strong mitotic index. CT scan also showed the appearance of several subcutaneous nodules of chest wall. This relapse was stabilized by 8 cycles of chemotherapy (gemcitabine-docetaxel regimen). Maintenance chemotherapy based on oral metronomic cyclophosphamide was started, but the disease worsened locally on April 2014, leading to palliative radiotherapy targeting the cutaneous and subcutaneous nodules of the right anterior chest wall. On September 2014, loco-regional progression involving the right anterior chest wall and the liver was documented on CT scan (Fig. 4b). Oral etoposide was started on October 2014. Despite treatment, the disease further worsened and the patient died on March 2015.

## Discussion

We report an exceptional case of soft tissue sarcoma arising in a refractory and locally advanced DT. As usually observed at the time of diagnosis of mammary DT [5], our patient was older than patients with abdominal location and the stage was advanced. Given the tumor volume, the initial treatment was medical, and during almost 2 years, the patient received successively NSAIDs, two regimens of hormone therapy, two regimens of chemotherapy and three targeted therapies without success. After 2 years of treatment, a suspect modification of the clinico-radiological aspect led to surgery. Pathological analysis of the operative specimen discovered a high-grade undifferentiated soft tissue sarcoma within the DT. Such pleomorphic sarcomas (ex-MFH, malignant fibrous histiocytoma) are typically deep-seated and aggressive tumors with both local and metastatic risks [12]. Four months after surgery, the patient showed locoregional relapse by the sarcoma, initially sensitive to gemcitabine-docetaxel, followed by multimetastatic progression refractory to several therapeutic lines, leading to the death 25 months after the surgery.

To our knowledge, this is the fourth case of sarcoma arising within a DT reported in the literature, and the first one in a breast DT. In the first case report published more than 50 years ago [9], a 19-year-old woman, without medical history, was operated for an inguinal 13-cm DT. During follow-up, she presented a local relapse treated with a total of four courses of radiotherapy over an 18-month period. Nine years after the last course, she presented a sudden increase in growth and cutaneous ulceration of the locally recurrent DT. Biopsy of this 15-cm mass identified a fibrosarcoma. Histopathologic examination of the surgical resection specimen showed a 5-cm high-grade fibrosarcoma surrounded by the DT. Six months after surgery, a local relapse of the fibrosarcoma occurred, requiring amputation. Following the amputation, several local recurrences were observed treated with surgery, then radiotherapy. Twenty months after amputation, the patient died. The second case [10] is a 53-year-old man, with significant history of Milroy’s disease (chronic hereditary lymphedema of unknown origin). He presented a non-tender 14-cm DT in the medial malleolar region, without any functional limitation, diagnosed by open biopsy. Given the infiltrative nature of the lesion and the minimal clinical symptoms, observation was decided. Two weeks after the biopsy, a small raised skin lesion appeared posterior to the open biopsy incision. A punch biopsy showed aspect consistent with DT. Three months later, a dramatic increase in the posterior mass occurred with a fungating and necrotic aspect; the radiological evaluation showed the previously-identified DT, as well as the new large fungating mass associated to one additional small area of abnormal signal within the DT. Biopsy of the fungating tumor identified a high-grade undifferentiated pleomorphic sarcoma, leading to below-knee amputation. The pathological analysis of the operative specimen showed the DT, which contained the two nodular masses corresponding to the undifferentiated sarcoma. Surgical margins were negative for sarcoma but microscopically positive for DT. Ten years after amputation, the patient was alive with no evidence of disease. The third case [11] is extracted from a series of eight patients in which the morphologic changes seen after radiotherapy in desmoid-type fibromatosis were retrospectively studied. The patient was a 12-year-old boy with initially a 9.5-cm DT of the proximal part of the upper limb treated by surgery. He presented a local relapse 36 months later that was treated with external radiotherapy (dose between 50 and 60 Gy). Sixteen years later, he underwent forequarter amputation because of a clinical progression of the tumor: the pathological analysis of the operative specimen demonstrated areas of fibrosarcoma with zonal necrosis, hypercellularity, severe nuclear atypia, and increased mitotic activity, adjacent to the locally recurrent DT. Three months after amputation, the patient was alive with no evidence of disease.

In summary, in all informative cases, the diagnosis of sarcoma was suspected by clinical modifications of the DT (volume increase, ulceration, multilobulated aspect) associated with radiological modifications with appearance of nodular masses within the DT. But these signs are not specific, and given the very exceptional character of sarcomatous degeneration of DT, the pre-therapeutic diagnostic confirmation was attempted in only one case, the diagnostic being done after surgery in the three other cases. Like for any soft tissue sarcoma, the treatment was margin-free monobloc surgery.

In pathophysiological term, in all cases, the sarcoma appeared within the DT and a specific risk factor for developing sarcoma was present: Milroy’s disease in one case [9], and radiotherapy in the three others ones with an interval between radiotherapy and sarcoma diagnosis of 9 years [9], 16 years [11], and 22 years (our case). In the two first cases, the radiotherapy was delivered in order to treat the DT, whereas in our case, it was delivered after surgery of breast cancer 20 years before the diagnosis of DT. In the absence of molecular analyses, it is not possible to know whether a common genetic mutation could explain the coexistence of DT and soft tissue sarcoma. Classically, the pleomorphic undifferentiated sarcomas display a complex genetic profile without specific structural alteration, whereas DTs display a simple genomic profile with specific mutation. The search for a common molecular alteration in both entities might benefit from the new next-generation sequencing techniques. Regarding the cell of origin, the embryologic derivation of the fibroblast (the mesoderm) renders it capable of giving rise, under appropriate oncogenic influences, to a wide range of proliferative manifestations [13] including for example aggressive fibromatosis, fibrosarcoma arising in a keloid, dermatofibrosarcoma protuberans, malignant fibrous histiocytoma, and also one case of aggressive fibromatosis progressing over 3 years to poorly differentiated sarcoma with widespread metastases, which may suggest a progressive increase in the malignant potential of the fibroblasts in the patient’s tumor.

## Conclusion

We report the fourth case of soft tissue sarcoma arising in a DT. This case reinforces the need for timely and accurate diagnosis when a desmoid tumor modifies its clinical or radiological aspect, notably in presence of associated risk factors such as history of radiotherapy. Given the exceptional nature of such diagnosis, case reports such as this one serve as the only reference for clinicians taking care of these patients.

## Consent

Written informed consent was obtained from the patient’s husband after her death for publication of this Case report and any accompanying images. A copy of the written consent is available for review by the Editor-in-Chief of this journal.

## Abbreviations

CT scan: computed tomography scan
DT: desmoid tumor
FAP: familial adenomatosis polyposis
HES: hematoxylin and eosin staining
IHC: immunohistochemistry
MFH: malignant fibrous histiocytoma
MRI: magnetic resonance imaging
NSAID: non-steroidal anti-inflammatory drug
*RRePS*: *Réseau de Référence en Pathologie des Sarcomes*
WHO: World Health Organization
